# Molecular mechanism underlying the di-uridylation activity of *Arabidopsis* TUTase URT1

**DOI:** 10.1093/nar/gkac839

**Published:** 2022-09-30

**Authors:** Qian Hu, Huiru Yang, Mingwei Li, Lingru Zhu, Mengqi Lv, Fudong Li, Zhiyong Zhang, Guodong Ren, Qingguo Gong

**Affiliations:** Department of Clinical Laboratory, The First Affiliated Hospital of USTC, Ministry of Education Key Laboratory for Membraneless Organelles & Cellular Dynamics, Biomedical Sciences and Health Laboratory of Anhui Province, School of Life Sciences, Division of Life Sciences and Medicine, University of Science and Technology of China, 230027 Hefei, P.R. China; State Key Laboratory of Genetic Engineering, Zhangjiang mRNA Innovation and Translation Center, School of Life Sciences, Fudan University, Shanghai 200438, China; Department of Clinical Laboratory, The First Affiliated Hospital of USTC, Ministry of Education Key Laboratory for Membraneless Organelles & Cellular Dynamics, Biomedical Sciences and Health Laboratory of Anhui Province, School of Life Sciences, Division of Life Sciences and Medicine, University of Science and Technology of China, 230027 Hefei, P.R. China; Department of Clinical Laboratory, The First Affiliated Hospital of USTC, Ministry of Education Key Laboratory for Membraneless Organelles & Cellular Dynamics, Biomedical Sciences and Health Laboratory of Anhui Province, School of Life Sciences, Division of Life Sciences and Medicine, University of Science and Technology of China, 230027 Hefei, P.R. China; Department of Clinical Laboratory, The First Affiliated Hospital of USTC, Ministry of Education Key Laboratory for Membraneless Organelles & Cellular Dynamics, Biomedical Sciences and Health Laboratory of Anhui Province, School of Life Sciences, Division of Life Sciences and Medicine, University of Science and Technology of China, 230027 Hefei, P.R. China; Department of Clinical Laboratory, The First Affiliated Hospital of USTC, Ministry of Education Key Laboratory for Membraneless Organelles & Cellular Dynamics, Biomedical Sciences and Health Laboratory of Anhui Province, School of Life Sciences, Division of Life Sciences and Medicine, University of Science and Technology of China, 230027 Hefei, P.R. China; Department of Physics, University of Science and Technology of China, Hefei, Anhui 230026, P.R. China; State Key Laboratory of Genetic Engineering, Zhangjiang mRNA Innovation and Translation Center, School of Life Sciences, Fudan University, Shanghai 200438, China; Department of Clinical Laboratory, The First Affiliated Hospital of USTC, Ministry of Education Key Laboratory for Membraneless Organelles & Cellular Dynamics, Biomedical Sciences and Health Laboratory of Anhui Province, School of Life Sciences, Division of Life Sciences and Medicine, University of Science and Technology of China, 230027 Hefei, P.R. China

## Abstract

In *Arabidopsis*, HESO1 and URT1 act cooperatively on unmethylated miRNA and mRNA uridylation to induce their degradation. Their collaboration significantly impacts RNA metabolism in plants. However, the molecular mechanism determining the functional difference and complementarity of these two enzymes remains unclear. We previously solved the three-dimensional structure of URT1 in the absence and presence of UTP. In this study, we further determined the structure of URT1 in complex with a 5′-AAAU-3′ RNA stretch that mimics the post-catalytic state of the mRNA poly(A) tail after the addition of the first uridine. Structural analysis and enzymatic assays revealed that L527 and Y592 endow URT1 with a preference to interact with purine over pyrimidine at the -1 RNA binding position, thus controlling the optimal number of uridine added to the 3′ extremity of poly(A) as two. In addition, we observed that a large-scale conformational rearrangement in URT1 occurs upon binding with RNA from an ‘open’ to a ‘closed’ state. Molecular dynamic simulation supports an open-closed conformational selection mechanism employed by URT1 to interact with RNA substrates and maintain distributive enzymatic activity. Based on the above results, a model regarding the catalytic cycle of URT1 is proposed to explain its di-uridylation activity.

## INTRODUCTION

Non-templated uridine addition to the 3′ end of RNA (RNA uridylation), aside from canonical poly(A) tails, has emerged as an important post-transcriptional RNA modification due to the fast development of RNA-sequencing technologies in recent years (1). 3′ uridylation generally serves as a degradation signal for both long and small RNAs no longer needed by the cell, balancing the dynamic equilibrium of RNA abundance and significantly impacting RNA metabolism (2,3). RNA uridylation is catalyzed by a class of template-independent terminal ribonucleotide transferases (TENTs) or terminal uridylyl transferases (TUTases) (4,5). In *Arabidopsis*, HEN1 SUPPRESSOR1 (HESO1) and URT1 (UTP:RNA uridylyltransferase) act cooperatively on unmethylated miRNA uridylation to trigger their degradation (6–9). HESO1 and URT1 also add U-tails to the 5′ cleavage product from miRNA-mediated AGO1 slicing (10,11). In both cases, HESO1 plays a major role. In addition, URT1 uridylates a subset of mRNAs with short poly(A) tails and is negatively involved in post-transcriptional gene silencing (12–15). Although a clear relationship has been established between uridylation catalyzed by these two TUTases and RNA turnover, the precise biochemical mechanisms of HESO1 and URT1 remain to be explored.

We recently determined the high-resolution structures of URT1 in the absence and presence of UTP (16). It consists of two canonical globular domains, the catalytic domain and the central domain, separated by a large catalytic groove where RNA substrate usually binds. The enzymatic domain structure of URT1 resembles those of other TUTases so far determined from other species and exhibited the highest similarity to that of *Schizosaccharomyces pombe* Cid1 (17–20). The UTP binding site of URT1 is almost identical to that of Cid1 (16). The only noticeable conformational difference of URT1 from other TUTases is that its catalytic groove is more open, suggesting better accessibility for RNA substrates.

As indicated above, URT1 can catalyze the 3′-uridylation of both miRNAs and mRNAs in plants. Tu *et al.* reported that URT1 and HESO1 act collaboratively on miRNAs *in vivo*, with a proposed model that URT1 mono-uridylates certain miRNAs for their further uridylation by HESO1 (8). On the other hand, Zuber *et al.* indicated that URT1 restored the 3′ extremity of deadenylated mRNA from an oligo(A) size >13–15 As to size distribution of poly(A + U) centered at 16 nt to allow the effective binding by Poly(A) Binding Protein (PABP) (13). In other words, about two uridines were averagely added to the deadenylated poly(A) tails of mRNAs by URT1. Although multiple studies showed that URT1 is capable of adding a long U-tail to the 3′-end of RNA under certain experimental conditions in vitro, the above lines of evidence suggested that URT1 functions as a TUTase that mainly catalyzes the mono- and/or di-uridylation on RNA substrates in vivo (8,9). Interestingly, as a structural homolog close to URT1, Cid1 was also reported to exhibit similar uridylation behavior, with predominant mono- and di-uridylation activities *in vivo* but showing robust activity of adding long U-tails in vitro (21,22).

HESO1, the functional paralog of URT1 in Arabidopsis, has been proven to add a long U-tail to the 3′-end of miRNAs both in vivo and in vitro (8). In addition, both HESO1 and URT1 contain no other known domain(s) except the enzymatic module (23,24). Therefore, it becomes intriguing to explore why, under the same physiological conditions, URT1 only adds a minimal number of Us (one or two) whereas HESO1 effectively extends U-tail to a longer size, especially considering all already known structures of TUTase are highly similar.

In this research, using an *in-vitro* transferase assay, we first identified that URT1 preferred to add two uridines at the 3′-end of poly(A). Furthermore, we determined the structure of URT1 in complex with a 5′-AAAU-3′ RNA stretch that mimics the post-catalytic state of the mRNA poly(A) tail after the addition of the first uridine. Structural analysis revealed that the URT1–AAAU interaction at -1 position is crucial for URT1 to discriminate between purine and pyrimidine, and further control the optimal number of uridine added to 3′ extremity of poly(A) as two. Site-directed mutagenesis of key residues followed by in-vitro transferase assays and in-vivo functional complementation tests validated this finding. In addition, we observed that a large-scale conformational rearrangement in URT1 occurs upon binding with RNA from an ‘open’ to a ‘closed’ state. Further molecular dynamic simulation supports an open-closed conformational selection model employed by URT1 to interact with RNA substrates and maintain distributive enzymatic activity. Our research reveals that the crucial structural factors determining the di-uridylation activity of URT1 may serve the effective functional reformation of URT1 and potentially lead to the illustration of the biological significance of dual TUTases (HESO1/URT1) in *Arabidopsis*.

## MATERIALS AND METHODS

### Cloning, expression and purification

The enzymatic domain of URT1 (residues 410–764) was amplified by PCR using synthetic gene as the template and was subcloned into pET28a vector with a 6 × His tag at the N-terminal site. All mutants were obtained by PCR and MutanBEST kit (Takara Bio Inc.), and verified by sequencing. Plasmids was transformed into *Escherichia coli* Gold (DE3) cells for expression. For overexpression of wild-type and mutant proteins, cells were cultured in LB medium at 37°C until OD_600_ reached 0.8–1.0 before isopropyl β-d-1-thiogalactopyranoside (IPTG) with a final concentration of 0.5 mM was added and then cultured at 16°C for 24 h.

The cells were collected by centrifugation and suspended by binding buffer (20 mM Bis–Tris, pH 6.0 and 1 M NaCl), followed by sonication lysis and centrifugation at 12 000 rpm for 30 min at 4°C. The supernatant was purified by Ni-NTA column (Qiagen) and further purified by Superdex™ 200 pg (16/60) (GE Healthcare). The purified proteins were then dialyzed against storage buffer (20 mM Bis–Tris, pH 6.0, 300 mM NaCl, 2 mM DTT and 5% glycerol) and concentrated to 3.5 mg/ml.

### Crystallization and data collection

For the crystallization of URT1 complexed with RNA, purified URT1 D547A mutant, whose catalytic activity was abolished (16), was concentrated to 3.5 mg/ml and then incubated with 5′-AAAU-3′ RNA stretch (synthesized from Takara Bio Inc.) at a molar ratio of 1:1.5. The crystals of URT1–AAAU were harvested in a sitting-drop vapor diffusion setup using a 1:1 ration of protein to reservoir at 20°C. The reservoir solution contains 0.1 M sodium HEPES, pH 7.5, 27% PEG 600. The crystals were cryogenically protected in their respective reservoir solutions supplemented with 25% (v/v) glycerol, and then rapidly frozen in liquid nitrogen. Diffraction datasets for all crystals were collected at Shanghai Synchrotron Radiation Facility (SSRF) Beamline 19U1.

### Structure determination and refinement

X-ray intensity data of the crystals were indexed, integrated, and scaled by HKL2000 package (25). The complex structure of URT1–AAAU was determined by using apo-form URT1 (PDB ID: 6L3F) as the search model (16). The model was built and refined using COOT (26) and Refmac5 (27) in the CCP4 package and Phenix (28). All images of the structures were prepared using PyMOL (http://www.pymol.org/).

### *In-vitro* nucleotide transferase assay

Purified URT1 wild type and its mutant were prepared for oligouridylation activity assay which was performed at 25°C in a buffer containing 20 mM Bis-Tris, pH 6.0, 100 mM NaCl, 10 mM MgCl_2_, 5 mM DTT and 5% (v/v) glycerol. 100 nM 13-nt poly(A) or its variants (synthesized from Takara Bio Inc.) with Cy5 probes labeled at the 5′ end was mixed with 50 nM protein and 1 mM UTP. The reaction was stopped at a series of time points (1, 3, 5, 10, 15, 30 min) by adding EDTA (final concentration 100 mM). The product was denatured at 95°C for 2 min, incubated on ice for 2 min, and analyzed with 20% denaturing polyacrylamide gels. RNA bands were imaged on a Typhoon FLA 7000 (GE Healthcare) by detecting Cy5, and quantified using ImageQuant TL (GE Healthcare). Rolling-ball algorithm was used for background subtraction.

The enzymatic assay in the presence of a trap (competitor) was performed by pre-incubation of URT1 with Cy5-labeled 13-nt poly(A) for 20 min on ice, after which a 100-fold molar excess of the same but unlabeled poly(A) was added as a trap at the same time as the reaction was initiated using 10 mM MgCl_2_ and 1 mM UTP. The reaction was stopped by the addition of EDTA (final concentration 100 mM) after incubation for 15 or 30 min at 25°C. The product was denatured at 95°C for 2 min, incubated on ice for 2 min, and analyzed with 20% denaturing polyacrylamide gels.

### Genetic transformation

Wild-type URT1 in the binary vector pMDC83 (*p35S::* URT1-GFP) was described previously (9). Site-directed mutations were introduced by overlap-extension PCR with mutagenic primers, followed by DpnI digestion and annealing. The resultant construct was transferred into the Agrobacterium strain GV3101 and was used to transform the *hen1-2 heso1-2 urt1-3* plants via floral dipping (29). T1 transgenic plants were selected on $\frac{1}{2}$ MS medium containing 25 μg/ml hygromycin and were transferred to the soil under long-day growth conditions (16 h light/8 h dark, ∼100 μmol m^−2^ s^−1^) at 22°C.

## RESULTS

### URT1 exhibits di-uridylation activity in vitro

**Figure 1.**
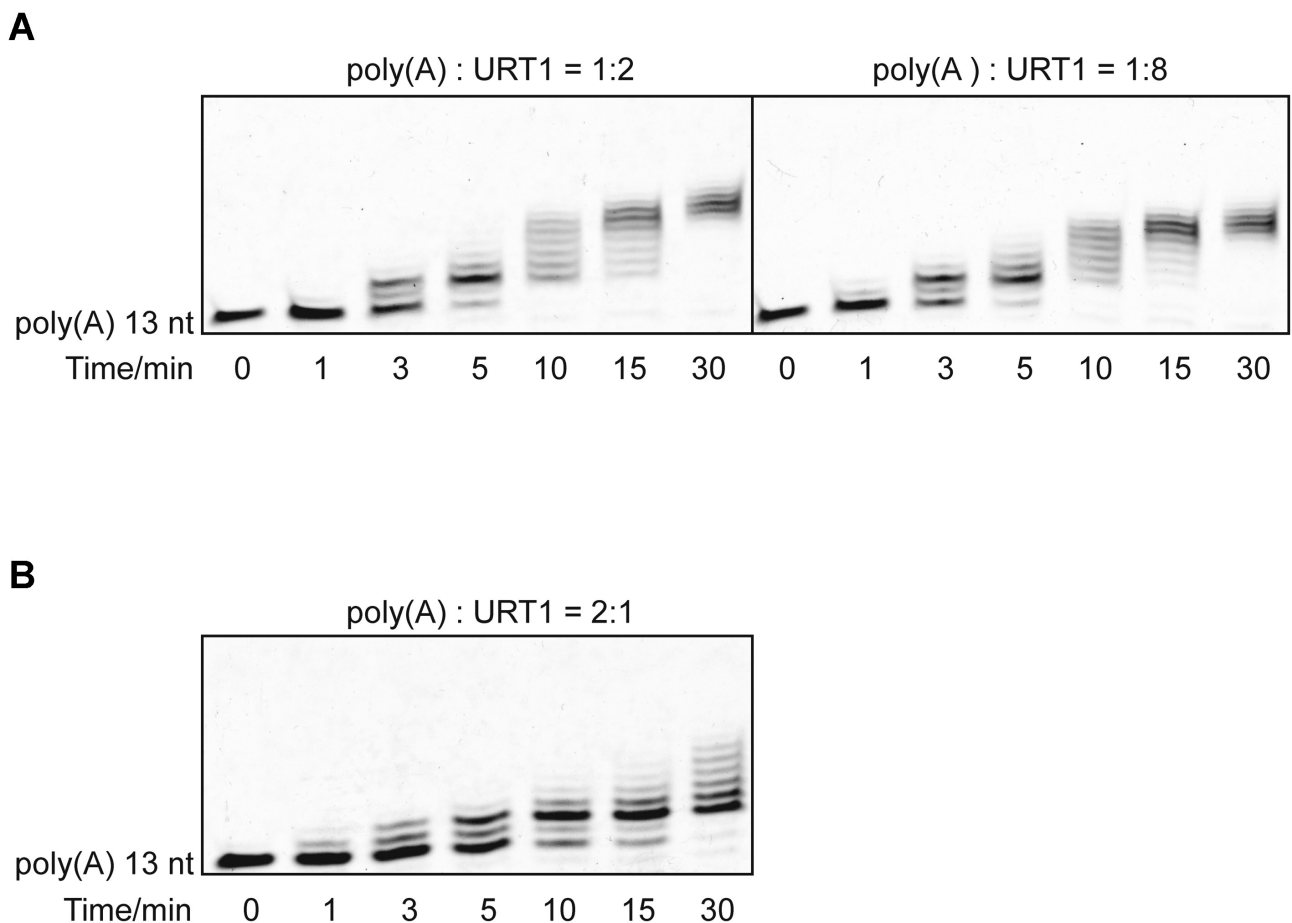
The core enzymatic domain of URT1 exhibits di-uridylation activity *in vitro*. (**A**) *In vitro* nucleotide transferase assay of 13-nt poly(A) by wild-type URT1. The molar ratios of poly(A) and URT1 are 1:2 (left) and 1:8 (right), respectively. (**B**) *In vitro* nucleotide transferase assay of poly(A) by URT1. The molar ratios of poly(A) and URT1 is 2:1.

URT1 has been reported to tail 3′-end of sRNAs and mRNAs with 1- or 2-nt uridines in vivo, while much longer U-tails added to RNA substrates are observed under in-vitro conditions (8,9). Although this apparent discrepancy in U-tail size can be simply explained by different experimental conditions and a much higher concentration of TUTase used in vitro, one might still wonder if there is any molecular mechanism in URT1 to regulate the number of uridines it added to the RNA substrates. To this end, we first utilized an in-vitro nucleotide transferase assay to investigate the URT1-induced uridylation of mRNA using a 13-nt poly(A) as the substrate, which is designed to represent the poly(A) tail of mRNA. Our initial time-course experiments showed that URT1 (410–764) could tail the poly(A) very efficiently when TUTase was much excessive in the experimental environment (poly(A): URT1 = 1:2 or 1:8) (Figure 1A). These experiments showed highly similar results that almost all RNA substrates are tailed after 10 min, and the U-tail with a length of >10-nt can be eventually added to 3′-end of poly(A) at the 30-min time point. Intriguingly, at the 3-min and 5-min time point, we did observe a noticeable accumulation of the poly(A) species with two added Us (poly(A)+2U) in the RNA ladders. To further confirm this result, we slowed down the uridylation reaction by changing the ratio of poly(A): URT1 to 2:1. Under the new experimental condition, persistent accumulation of poly(A)+2U bands was clearly observed at different time point from 5-min to 30-min, although long U-tails were still formed when the reaction time was longer than 10 min (Figure 1B), indicating that URT1 does prefer to add two Us to the 3′-end of poly(A) tail of mRNA. It is reasonable to assume that, under experimental conditions closer to the physiological scenario in which the concentration of TUTase is much lower, poly(A)+2U will become the dominant RNA species in the URT1-mediated uridylation event.

In addition, a possibility raised by the accumulation of poly(A)+2U bands in our assays is that the addition of the first two uridines by URT1 could proceed in a processive manner while further uridylation is distributive. To testify this possibility, we re-performed the enzymatic assay in the presence of a trap (30). Specifically, after pre-incubation of URT1 with labeled 13-nt poly(A), a 100× molar excess of unlabeled poly(A) was added as a trap at the same time as the metal ion and UTP. The reaction was stopped by the addition of EDTA after incubation for 15 or 30 min. Our results showed that (Supplementary Figure S1), for both 15- and 30-min reactions, a significant slow-down of the tailing process and both bands representing poly(A) added with one and two uridines were observed, suggesting the uridylation activity of URT1 for the first two uridines is mainly distributive. We also conducted same experiments on R531A which is a mutant designed to disrupt the closed conformation of URT1 and further affect its association with RNA (see later), and similar but weaker results were obtained. Together, our results suggested that, not only *in vivo* but also *in vitro*, URT1 shows a di-uridylation distributive activity toward RNA substrates.

### Protein-RNA interaction details in URT1–AAAU

**Figure 2.**
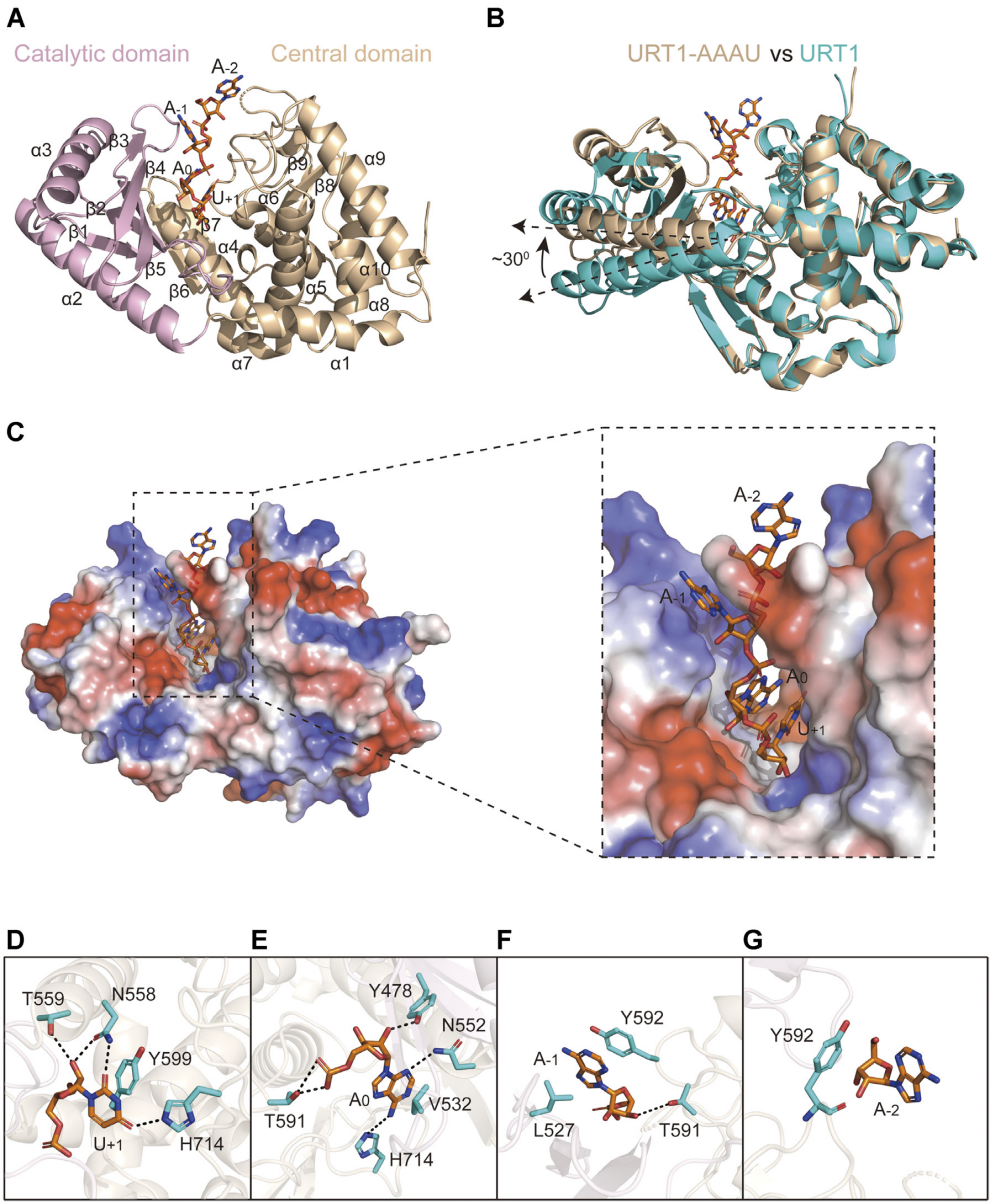
The crystal structure of URT1 enzymatic domain in complex with RNA stretch 5′-AAAU-3′. (**A**) Cartoon representation of URT1–AAAU complex structure. The catalytic and central domains are shown in pink and wheat, respectively. The RNA stretch 5′-AAAU-3′ is shown in stick model. (**B**) The structural superimposition of apo-form (teal) and RNA-bound (wheat) URT1. (**C**) The overview of 5′-AAAU-3′ lying along the catalytic groove of URT1. URT1 is shown in electrostatic surface potential, and RNA stretch is shown in stick model. (*Inset*) A close-up of the engagement of 5′-AAAU-3′ into the catalytic groove. (**D–G**) URT1–AAAU interaction details at four different nucleotide binding sites (−2, −1, 0 and +1 positions). Nucleotides and surrounding residues are shown in orange and cyan sticks, respectively. Hydrogen bonding interactions are all indicated as black dashed lines.

We previously determined the structures of URT1 (410–764) and its complex with UTP using X-ray crystallography (16). URT1 with and without UTP exhibit an almost identical fold with the electron densities of a few regions (410–424, 692–700 and 756–764) are missing, probably due to local high structural flexibilities. To further explore the URT1-mRNA recognition, we co-crystalized the URT1 (410–764) with a 5′-AAAU-3′ RNA stretch and obtained their complex structure at 1.8 Å resolution (Figure 2A, Table 1). In this structure, RNA bound URT1 in a canonical way with 3′-U sitting in the UTP binding site (+1 position) of URT1, and three other adenines sequentially occupied the 0, –1 and –2 RNA-binding positions along the catalytic groove (Figure 2C). Unexpectedly, different from our and other previous researches in which the structures of apo- and RNA-bound forms of TUTases are basically the same (19,20,31), URT1 experienced a large conformational rearrangement upon the RNA binding, with its entire catalytic domain rotated by ∼30° from its original position in apo-form (open state) toward to the central domain, about the joint region of these two domains, to form a more compact structure (closed state) (Figure 2B). The molecular mechanism of this open-to-closed conformational transition will be discussed in detail later.

**Table 1.** Data collection and refinement statistics

**Data collection**	URT1–AAAU
Beamline	19U, SSRF
Space group	*P*2_1_2_1_2_1_
PDB code	7XS4
Wavelength (Å)	0.97929
Resolution (Å)	40.00–1.85 (1.88–1.85)^a^
Cell dimensions	
*a*, *b*, *c* (Å)	63.16, 63.26, 81.39
α, β, γ (°)	90, 90, 90
Unique reflections	28 694 (2803)
Completeness (%)	100.00 (100.00)
Redundancy	10.6 (10.9)
*I*/σ*I*	24.08 (2.5)
*R*_merge_ (%)	7.8 (69.6)
CC_1/2_	1.00 (0.887)
CC*	
**Refinement**	1.00 (0.970)
*R*_work_ (%)	17.31
*R*_free_ (%)	20.15
No. of atoms	
Protein	2583
RNA	83
Water	269
Average *B* factors (Å^2^)	
Protein	29.52
RNA	43.75
Water	38.22
Root mean square deviations	
Bond lengths (Å)	0.004
Bond angles (°)	0.64
Ramachandran plot	
Favored (%)	98.15
Allowed (%)	1.85
Disallowed (%)	0.00

^a^Values in parentheses are for highest-resolution shell.

**Figure 3.**
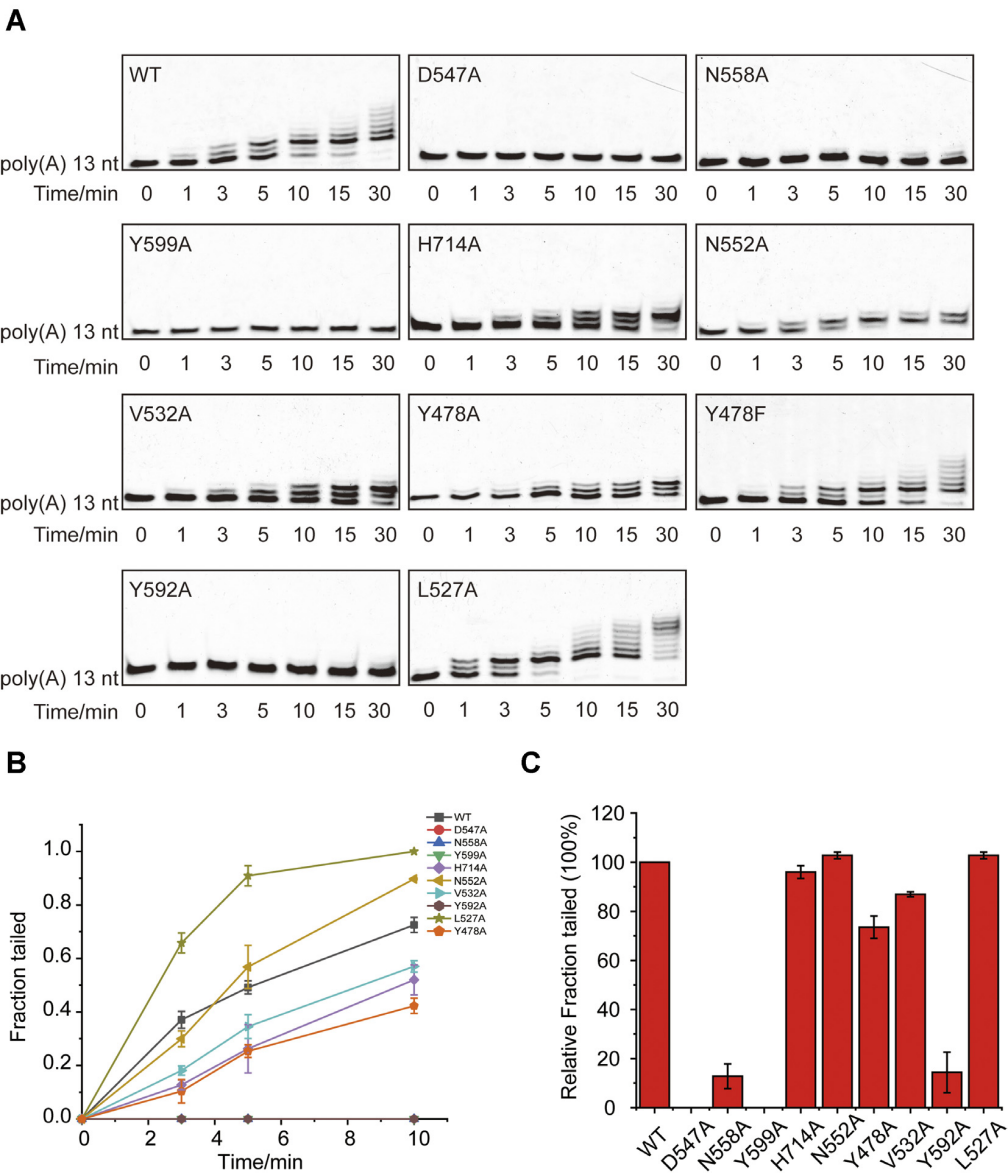
Protein-mRNA interaction verification in URT1–AAAU. (**A**) *In vitro* nucleotide transferase assay of 13-nt poly(A) against wild-type URT1 and different URT1 mutants including D547A, N558A, Y599A, H714A, N552A, V532A, Y478A, Y478F, Y592A and L527A. (**B**) Quantification of three independent replicates of the experiment shown in Supplementary Figure S5. Data presented as mean ± SD. (**C**) The comparison of tailing efficiency at 30-min time point. The columns represent the tailed fractions of poly(A) in the assay. The fraction tailed by wild-type URT1 is normalized to 100%.

At the +1 position, the terminal U (U_+1_) of RNA stretch adopted very similar conformations for its base and sugar ring as those of the UTP molecule in the URT1-UTP structure (PDB ID 6L8K) (Supplementary Figure S2), indicating that our URT1–AAAU structure is a reasonable representation of the post-catalytic state of the addition of the first 3′ U. Major protein–RNA interactions at this site were highly conserved among TUTases from different species with the side-chains of URT1 N558 and H714 created direct hydrogen bonds with the carbonyl groups (O2 and O4) of U_+1_ base, respectively, and the base conformation was further stabilized by the hydrophobic stacking interactions from Y599 (Figure 2D). For the sugar ring moiety, the side-chain groups of N558 and T559 formed two hydrogen bonds with the 2′-hydroxyl group of U_+1_. We then mutated N558, Y599 and H714 to alanine to evaluate the effects of the destruction of URT1 interactions with the U_+1_ base on URT1 function. Our *in-vitro* nucleotide transferase assay showed that N558A and Y599A almost abolished the enzymatic activity of URT1, similar to the catalytic-dead mutant D547A, which served as a negative control in this research (16), while the U-tailing capacity of H714A was partly reduced (Figure 3). These results confirmed that N558 and Y599 are essential residues in stabilizing the conformation of UTP for effective uridylation reaction, and H714 may be less important in this issue but play a role in discriminating uracil over other three types of nucleotides as suggested by previous research (16).

**Figure 4.**
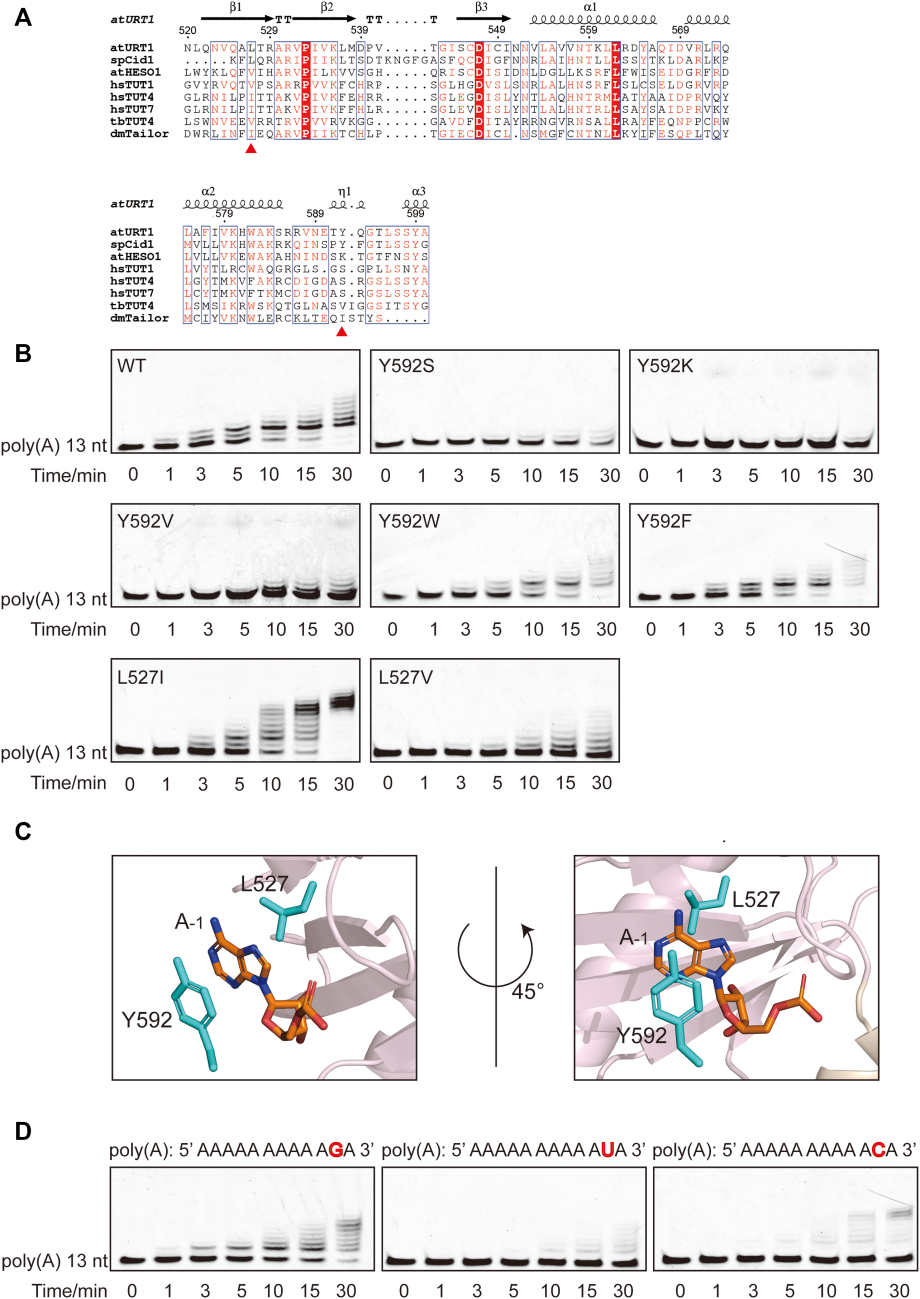
L527 and Y592 help URT1 to discriminate between purine and pyrimidine at the -1 position. (**A**) Multiple sequence alignments of URT1 with the TUTases from different species, including *sp*Cid1 from *S. pombe* (UniProtKB entry O13833), *at*HESO1 from *Arabidopsis* (Q5XET5), *hs*TUT1, *hs*TUT4, *hs*TUT7 from *H. sapiens* (Q9H6E5, Q5TAX3, Q5VYS8, respectively), *tb*TUT4 from *Trypanosoma brucei* (Q381M1) and *dm*Tailor from *Drosophila melanogaster* (Q9VI58). The secondary structure of the URT1 enzymatic domain is shown above the sequences. L527 and Y592 are highlighted by red triangles below the sequences. Strictly conserved residues and highly conserved residues are colored red background and red font, respectively. (**B**) *In vitro* nucleotide transferase assay of poly(A) against wild-type URT1 and different URT1 mutants including Y592S, Y592K, Y592V, Y592W, Y592F, L527I and L527V. (**C**) Close-up view of the interaction between URT1 and RNA at the –1 position from two angles. The adenosine at the –1 position is depicted as orange sticks, while surrounding residues are shown in cyan sticks. (**D**) *In-vitro* nucleotide transferase assay of 13-nt poly(A) mutants (the second A in the 3′ terminal is mutated to G, U and C, respectively) against wild-type URT1. The sequences of mutants are displayed at the top of gel result. Mutated nucleotides are shown in red font.

In the URT1–AAAU complex, the 0 nucleotide-binding position was occupied by an adenine (A_0_) representing the 3′ extremity of the mRNA poly(A) tail. Among the URT1 residues surrounding A_0_, N552 provided the specific contact with the adenine by forming a hydrogen bond between its side-chain group and purine N3, while V532 made a hydrophobic interaction against the base (Figure 2E). Both N552 and V532 are conserved residues and undertake similar tasks in all TUTases (Figure 4A). We then mutated these two residues to alanine (N552A and V532A) and estimated their contributions to the URT1 function using the transferase assay. Intriguingly, our results indicated that the enzymatic activity of URT1 remained almost intact in N552A and was slightly perturbed by V532A (Figure 3). Nevertheless, both mutations impaired the URT1 function by decreasing its ability in U-tail extension, and only two Us were added to the 3′-end of poly(A), even after longer incubations (i.e. 30 min) (Figure 3A). Other protein–RNA interactions at the 0 position included the hydrogen bond formed between Y478 side-chain and the 2′-hydroxyl group of A_0_ and the hydrogen bonds formed between T591 and the phosphate group (Figure 2E). We also generated Y478 mutations (Y478A and Y478F) and applied them to the transferase assay. Y478A exhibited a very similar effect on the URT1 tailing profile as N552A, while Y478F largely retained the enzymatic activity and tailing pattern of URT1 (Figure 3), suggesting protein–RNA interactions at the 0 position are more and less associated with the elongation of U-tails.

Unlike the +1 and 0 nucleotide-binding sites, URT1 possesses a group of less conserved residues for RNA recognition at the –1 position (Figure 4A), where the protein–RNA interactions are dominated by hydrophobic stacking. The adenine at this site (A_–1_) was perfectly sandwiched by the side-chain groups of Y592 from the central domain and L527 from the catalytic domain (Figure 2F). Our *in-vitro* assay showed that the destruction of Y592 stacking on A_–1_ base using the Y592A mutation abolished the enzymatic activity of URT1, whereas L527A showed almost no impacts on its tailing pattern (Figure 3), indicating that the π–π stacking between Y592 and A_–1_ is the most crucial interaction at this site. In addition, T591 also made a non-specific interaction with A_–1_ by forming a hydrogen-bonding interaction with its sugar ring to further consolidate the protein–RNA recognition. At last, the 5′ adenine of 5′-AAAU-3′ (A_–2_) was located at the exit of the catalytic groove of URT1, making no obvious interactions with the protein (Figure 2C and G). Its conformation is possibly stabilized by the crystal packing.

### L527 and Y592 help URT1 to discriminate between purine and pyrimidine at the –1 position

To explore whether there exists any structural determinant(s) in URT1 responsible for its di-uridylation activity, we re-analyzed the URT1–AAAU complex structure. URT1 distinguishes itself from other TUTases with reported protein–RNA complex structures (*dm*Tailor, *hs*TUT7, *sp*Cid1, and *tb*TUT4) mainly through its RNA recognition mode at the –1 position (19,20,32,33). As two key residues in stabilizing the conformation of A_–1_ base, Y592 is not conserved across different species, and L527 is also not strictly conserved according to the sequence alignment (Figure 4A). To further explore the functional significance of Y592, we mutated it to different types of residues existing in other TUTases, including serine, lysine, and valine (Y592S, Y592K and Y592V), for in-vitro transferases assay. All these mutants showed similar experimental phenomena as Y592A, with almost abolished or largely reduced enzymatic activity (Figure 4B). By contrast, two aromatic mutants (Y592W and Y592F) retained the catalytic activity and tailing pattern of URT1 (Figure 4B), confirming that the aromatic stacking interaction from Y592 is necessary for URT1 to maintain its catalytic function toward RNA substrates. In addition, we mutated L527 to alanine or other similar hydrophobic residues (L527I and L527V) for tailing assay. Although L527A showed almost no effect on the U-tailing ability of URT1 (Figure 3A), other mutants exhibit different results. The transferase activity of L527V was evidently reduced while that of L527I dropped very slightly (Figure 4B). Considering valine has a shorter side-chain while leucine and isoleucine are similar in the length of side-chain, it may not be surprising that L527V can not stack with adenine properly and cause an overall reduced enzymatic activity, whereas L527I maintains a similar activity as wild-type URT1.

At the –1 position, we noticed that both the side-chains of L527 and Y592 stacked exactly with the pyrimidine ring of A_–1_ but not its imidazole ring (Figure 4C). If we replaced A_–1_ with uridine in our structure, the side-chains of L527 and Y592 would be too far from the uracil to form effective stacking interactions (Supplementary Figure S3). We then speculated that the URT1-RNA recognition would be affected if adenine is replaced by uracil at this site. To verify this hypothesis, we generated three 13-nt poly(A) mutants with the adenine adjacent to 3′-A is substituted by guanine, uracil, and cytosine (A2G, A2U and A2C), respectively, and tested them using a tailing assay. Clearly, our results showed that both A2U and A2C are weakly tailed by URT1 whereas A2G is properly tailed in a pattern very similar to poly(A) but with somewhat lower efficiency (Figure 4D). These results are consistent with our above speculation and suggest a conclusion that L527 and Y592 help URT1 to establish a preference for purine over pyrimidine at the –1 nucleotide-binding position.

Interestingly, compared with wild-type URT1, both L527I and L527V mutants showed no appreciable accumulation of RNA species with two added Us during the entire tailing process. We proposed that the minor perturbation through substitution of leucine by isoleucine or valine could fine-tune the nucleotide-binding preference at the –1 position so that uridine has a better chance to be incorporated into the polyuridylation chain.

### URT1 employs an open-closed conformational selection mechanism for RNA binding

As shown above, URT1 experienced a large-scale motion of its catalytic domain toward the central domain upon binding the 5′-AAAU-3′ RNA stretch (Figure 2B). Except for the global motion, the conserved substrate specificity loop (SSL) (20) of the catalytic domain also underwent a local structural rearrangement. Consequently, the side-chain groups of L527 and V532 shifted by 9.7 and 6.7 Å, respectively, from their positions in the apo form to properly stack with bases of A_-1_ and A_0_. More strikingly, the R531 from the SSL loop largely re-orientated its side chain with the end group of side chain shifted by 16.9 Å to form a 2.6 Å hydrogen-bonding interaction (salt bridge) with D700 from the central domain (Supplementary Figure S4). To testify if this inter-domain interaction is important for URT1 function, three mutations (R531A, D700A, R531A/D700A) were prepared for in-vitro activity assay. Our results (Supplementary Figure S5) showed that both R531A and R531A/D700A have a largely reduced enzymatic activity, suggesting that R531 contributes to URT1 function probably through maintaining the closed conformation for RNA binding. The catalytic activity of D700A, however, remains almost unaffected. We reconciled the above results that D700 locates within a highly flexible loop (the electron densities of this loop are totally missing in apo-form URT1), and other residues (such as N699) within this loop may still be able to form hydrogen-bond interaction with R531 even when D700 is substituted with alanine.

**Figure 5.**
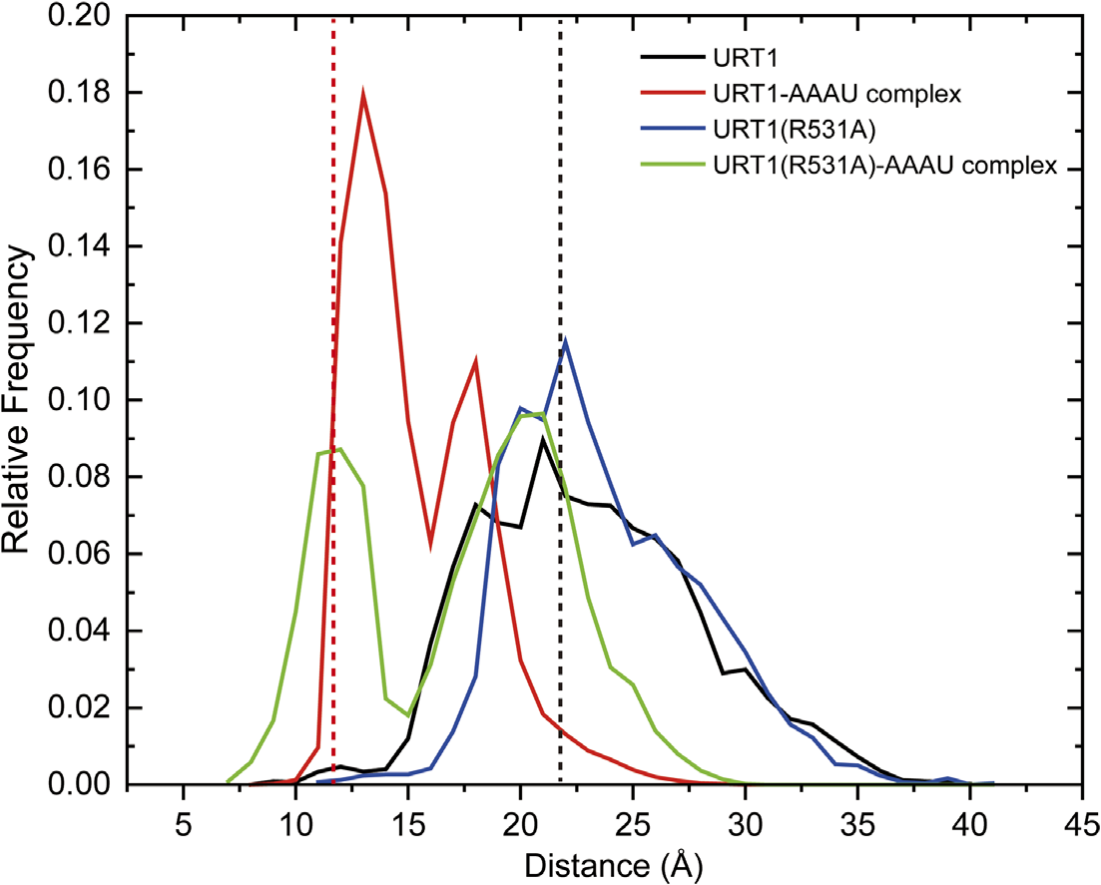
URT1 employs an open-closed conformational selection mechanism for RNA binding. The open/close distribution from aMD simulations of apo-form and RNA-bound URT1 (wild-type and R531A). The open/close is measured by the C_α_–C_α_ distance between R531 (or A531) and D700. The dashed lines indicate the distances of the closed (red and green) and the open (black and blue) crystal structures.

To further investigate whether URT1 employs conformational selection or induced fit as its RNA binding/catalytic mechanism, we performed the accelerated molecular dynamics (aMD) simulation of the URT1 system (see Supplementary Methods and Materials). Since the residues 692–700 (DWTRRVGND) and residues G689-N699 are missing in the crystal structures of apo-form URT1 and URT1–AAAU complex, we have modeled them by PyMOL (http://www.pymol.org/) for further simulation. To obtain statistically meaningful results, we performed three independent 2-μs aMD simulations for apo-form URT1 and URT1–AAAU, respectively. From each simulation, a trajectory containing 2000 conformations extracted every 1 ns were used for analysis. The three aMD trajectories were combined, and thus the 6000 conformations were used for analysis. The C_α_–C_α_ distance between R531 and D700 was calculated to measure the open/close of URT1 conformation. Figure 5 shows the distance distribution of URT1 and URT1–AAAU. In URT1, the major peak around 21.7 Å is the open state. Our simulations can sample conformations even more open than the experimental structure, with a population of 61.2% from 21.8 to 40.0 Å. It has been found that the apo form can also reach a partially closed state, with a minor peak around 18.0 Å. In URT1–AAAU, the major peak around 11.7 Å is the closed state, and the population is 58.0% from 9.0 to 16.0 Å. The minor peak is also around 18.0 Å as that of the open state, implying a possible intermediate state of URT1.

From our simulations, the apo-form URT1 is mainly in an open state, but it can occasionally sample the closed state with a population of 3.3% from 9 to 16 Å. Although URT1–AAAU is mainly in a closed state, it can sample the open state with a population of 10.2% from 21.8 to 30.0 Å. That is to say, there is an overlapping region of distance distribution between apo-form and RNA-bound URT1. Therefore, our results indicated that a dynamic conformational equilibrium exists between the open and closed states for the URT1 system, and RNA binding may shift the equilibrium from the open to the closed state via a ‘conformational selection’ mechanism.

In addition, we performed a parallel simulation on R531A mutant to analyze its effects on URT1 conformations (Figure 5). In URT1(R531A), the major peak is around 21.7 Å, with a population of 70% from 21.8 to 41.8 Å. The population is only 1.0% from 11.3 to 16.0 Å. In the URT1(R531A)-AAAU one peak is around 20 Å, and the population is 44% from 21 to 30 Å. The other peak is around 11.7 Å, and the population is the 36.0% from 7.3 to 16 Å. Our results indicated that, by mutating R531 to alanine, apo-form URT1 can sample more open states while the population of the open state in RNA-bound URT1 becomes more dominant than that of the closed state, confirming R531-participated inter-domain interaction is important in stabilizing URT1 conformation in the closed state.

Yates *et al.* had previously reported two apo-form crystal structures of *Schizosaccharomyces pombe* Cid1, one open and one closed, similar to the two conformations we obtained for URT1 (34,35). Given that Cid1 and URT1 are structural homologs with the highest similarity among all TUTases with known structures, both studies supported a conformational selection model used by URT1 and maybe Cid1 in their RNA substrate recognition.

### URT1–AAAU interaction residues are crucial for URT1 function *in vivo*

**Figure 6.**
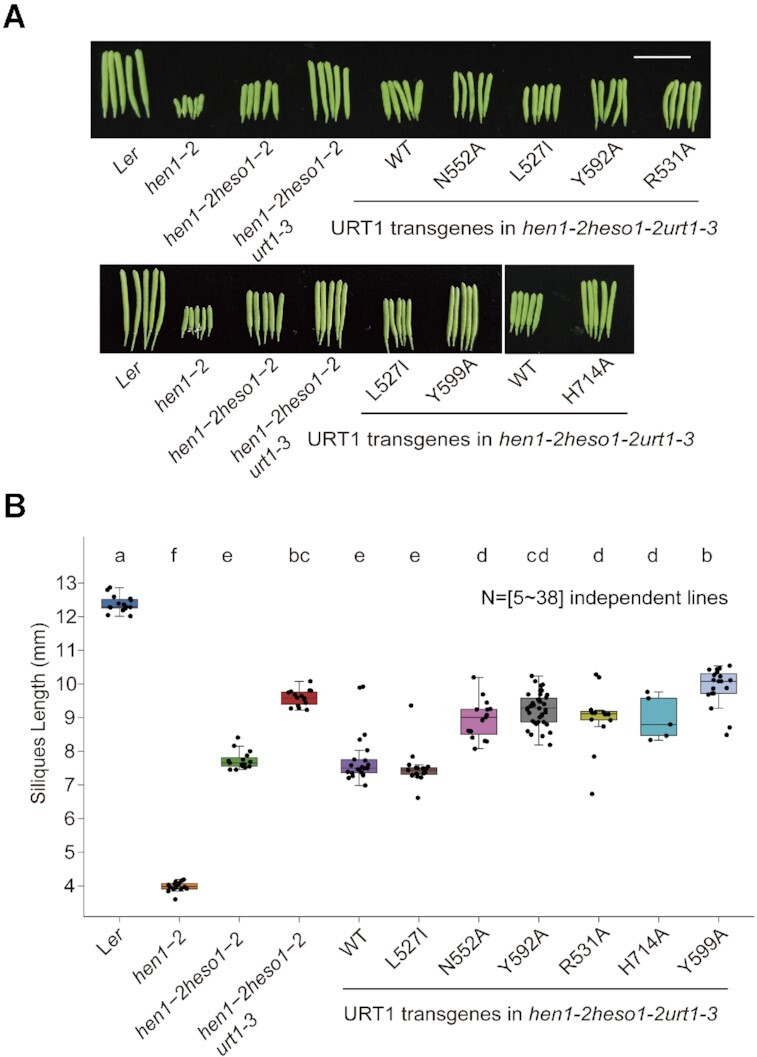
URT1–AAAU interaction residues are crucial for URT1 function in vivo. (**A**) Fully-expanded siliques of different genotypes. For URT1 transgenes, siliques from representative T1 transgenes were shown. Scale bar = 1cm. (**B**) Distributions of silique length in different genotypes. Circles represent individual control plants or independent T1 transgenes. For each individual, the average length of at least six fully-expanded siliques was used for the plot. Letters indicate statistically significant groups (ANOVA test, *P* < 0.05).

Neither the *urt1* single mutant nor *hen1 urt1* mutant has altered developmental defects as compared with their relative controls (i.e. Wt and *hen1*, respectively) (7). To assess the in vivo functions of URT1 residues, we took advantage of the *hen1-2 heso1-2 urt1-3* triple mutant, which has longer siliques and better fertility than *hen1-2 heso1-2* (9). Wild-type and various mutant *URT1* constructs driven by the constitutive 35S promoter (*p35S*::URT1-YFP) were individually transformed into *hen1-2 heso1-2 urt1-3*. For each construct, multiple independent T1 plants were examined. As a control, wild-type URT1 restored the silique length to the *hen1-2 heso1-2* level, but could not further reduce it (Figure 6) (9). Consistent with in vitro results, the U_+1_ interacting mutants (H714A/Y599A), the A_0_ binding mutants (N552A), the A_-1_ recognition mutants (Y592A), and the conformation change mutants (R531A) were significantly impaired or completely abolished rescuing the silique length phenotype. Y599A even had longer siliques than *hen1-2 heso1-2 urt1-3*. Considering that URT1-3 was not a null allele (9), this could be potentially caused by a dominant-negative effect. On the other hand, the L527I substitution fully rescued the *hen1-2 heso1-2 urt1-3* phenotype. These results strongly suggest that residues involved in –1, 0, +1 position binding are crucial for their proper functions.

## DISCUSSION

TUTases are originally evolved from canonical poly(A) polymerases (PAPs) and dedicated to catalyzing the uridylation of RNA substrates. Among all identified TUTases from different species, *Schizosaccharomyces pombe* Cid1 and *Arabidopsis* URT1 add a minimal number of uridines (one or two) to the 3′-ends of mRNAs or miRNAs in vivo, while others are often associated with the insertion of a polyuridylation chain to the RNA substrate (8,9,13,21,22,36). In addition, mammalian TUT7 and TUT4 have been reported to perform a Lin28-induced switch between two functional modes (mono-uridylation and oligo-uridylation), either promoting biogenesis of let-7 microRNA or marking it for degradation (19,37,38). In this research, using an in-vitro transferase assay, we confirmed that URT1 exhibits an intrinsic preference for di-uridylation activity toward mRNAs. Further structural analysis revealed that the hydrophobic protein–RNA stacking interactions contributed by L527 and Y592 at the -1 RNA binding site of URT1 are crucial factors in regulating the number of the uridines added to mRNA. The sequence alignment of URT1 with other TUTases indicates that L527 is a less conserved residue and Y592 is not conserved except in Cid1 (Figure 4A). Although the complex structure of Cid1 with an RNA substrate is absent, it is likely that Cid1 L135 and Y205 undertake the same tasks as their equivalent residues L527 and Y592 in URT1, given that Cid1 also prefers to add few uridines to RNA substrates under the physiological conditions. On the other hand, in HESO1, URT1 L527 and Y592 are substituted with valine and positively charged lysine (V110 and K175), which might help explain why HESO1 is capable of adding more uridines to RNA than URT1, although they are closely related functional paralogs in *Arabidopsis*. In fact, the examination of other available complex structures of TUTase with RNA substrates shows that direct and water-mediated hydrogen bonds dominate protein–RNA interactions at the –1 site in *dm*Tailor and *hs*TUT7 (19,20), different from the theme of hydrophobic interaction in URT1. In addition, the structures of *Tribulus terrestris* CutA in the apo form and its complex with A3 RNA have been recently reported (39). Intriguingly, although CutA also possesses a pair of hydrophobic residues V372 and F438 (equivalent to URT1 L527 and Y592), no stacking interactions are formed between CutA and A3 at the –1 position due to the totally different orientation of adenine from that in URT1–AAAU (Supplementary Figure S6). Whether CutA V372 and F438 perform similar function as their corresponding residues in URT1 needs further research. In summary, from an aspect of evolution, URT1 probably inherits the di-uridylation activity from Cid1, whereas HESO1 evolves divergently to obtain the capacity of polyuridylation, which is further stabilized in higher species.

Although both L527 and Y592 participate in the URT1–RNA recognition at the -1 position, their specific contributions are different. Our complex structure and enzymatic assay indicate that Y592 is mainly responsible for the protein–RNA interaction through aromatic π-π stacking, while L527 seems to play a role in fine tuning the tailing pattern. Compared with wild-type URT1, both L527I and L527V mutants exhibit a modified tailing process without the noticeable accumulation of di-uridylated RNA species. We wonder if L527I, which maintains a comparable enzymatic activity as wild-type URT1, is capable of adding more uridines (>2) at the 3′ end of RNA substrates in *Arabidopsis*. In fact, in-vivo studies indicated that, among all the mutants we tested, L527I exhibited the strongest effect in rescuing the *hen1-2 heso1-2 urt1-3* phenotype (Figure 6), consistent with our in-vitro observation that L-to-I substitution barely affects the enzymatic activity of URT1. We noticed that the average silique length of L527I is slightly shorter than that of wild type, although this difference is not statistically significant (Figure 6). Given that the corresponding residues of L527 in TUTases from animal species are all isoleucine, our results suggest that L527I variant may help URT1 to gain the activity of polyuridylation in vivo. Further investigations based on this site in combination with the Y592 site may lead the way toward reformation the URT1 function.

**Figure 7.**
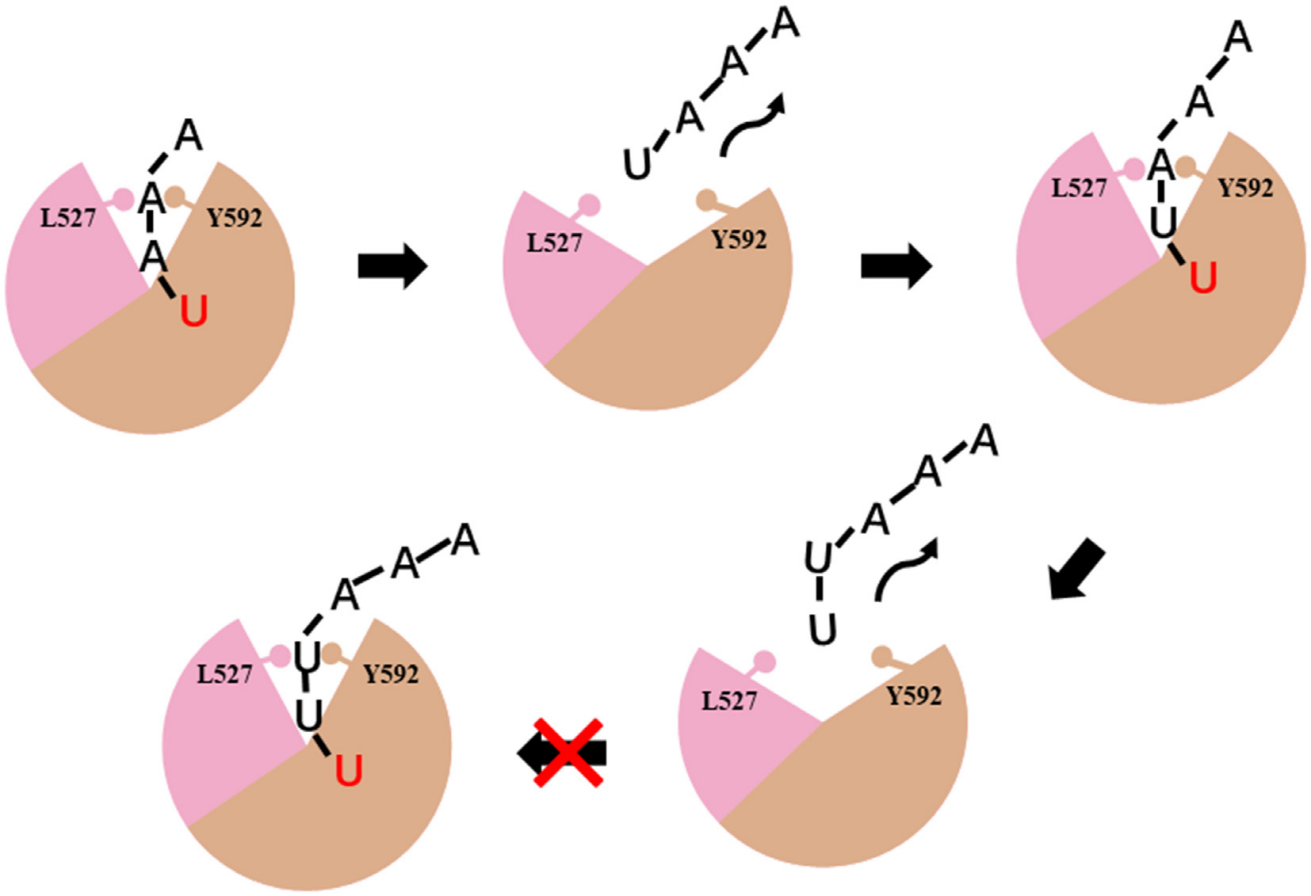
Proposed model of catalytic circles explains the di-uridylation activity of URT1. The catalytic and central domains are shown in pink and wheat, respectively. The lollipop shapes represent amino acid residues, with pink representing L527 and wheat representing Y592.

At last, a model regarding the catalytic cycle of URT1 is proposed to explain its di-uridylation activity, based on our findings in this research (Figure 7). First, the poly(A) tail of an mRNA substrate is fused with one uridine by URT1 and the product (poly(A) + U) is then released from the enzyme. Next, poly(A) + U will re-interact URT1 for the addition of the second uridine at its 3′-end. During the first two catalytic steps, L527 and Y592 interact with adenine at the –1 position. However, when poly(A) + 2U binds URT1 for the addition of the third uridine, L527 and Y592 will have to deal with the non-favorable uridine for this RNA binding site, and the addition of more uridines will become much less efficient than that of first two uridines due to the relatively weak stability of URT1–RNA interaction. In addition, during the catalytic cycles, an open/close conformational selection mechanism is employed by URT1 to interact with an RNA substrate, in which the open state of URT1 is probably a favorable conformation for initial engagement of the RNA substrate, and then conformational equilibrium shifts toward to the closed state which stabilizes the interaction between URT1 and RNA for the fulfillment of the enzymatic reaction. Upon the release of RNA product, the conformational equilibrium shifts back to the open state for the next-round reaction.

## DATA AVAILABILITY

Coordinate and structure factor for URT1–AAAU complex have been deposited in the Protein Data Bank under accession code 7XS4.

## Supplementary Material

gkac839_Supplemental_File
