# Supplementary material for: Molecular mechanism underlying the di-uridylation activity of Arabidopsis TUTase URT1
Source: Nucleic Acids Res. 2022 Sep 30;50(18):10614–25. doi: 10.1093/nar/gkac839 (PMC9561377; doi:10.1093/nar/gkac839)
Supplement: gkac839_Supplemental_File [file gkac839_supplemental_file.pdf]

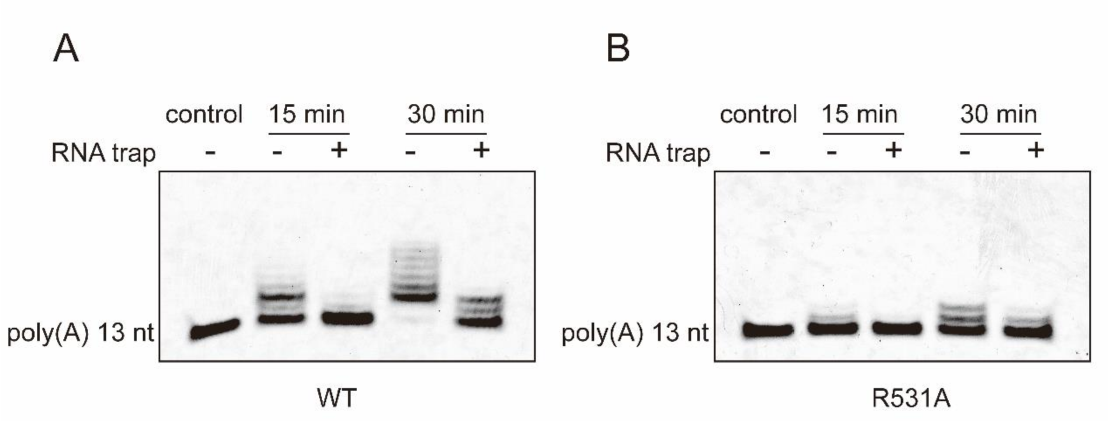

Figure S1. In vitro nucleotide transferase assay of Cy5-labeled poly(A) by wild-type URT1 (A) or R531A mutant (B) in the presence (+) of a 100-fold molar excess of the same but unlabeled poly(A) as a trap.

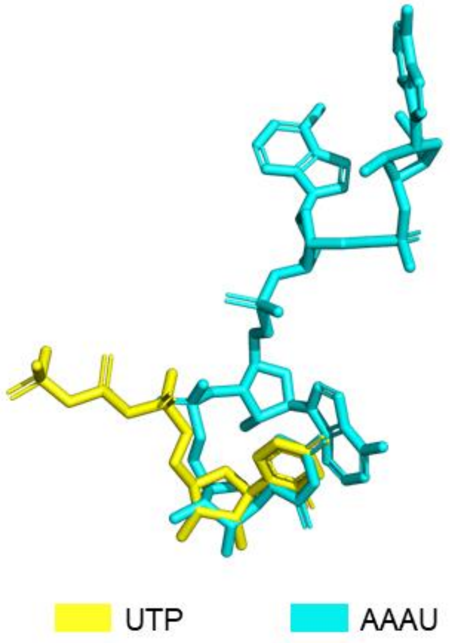

Figure S2. The structural superimposition of UTP (yellow) and 5'-AAAU-3' (cyan).

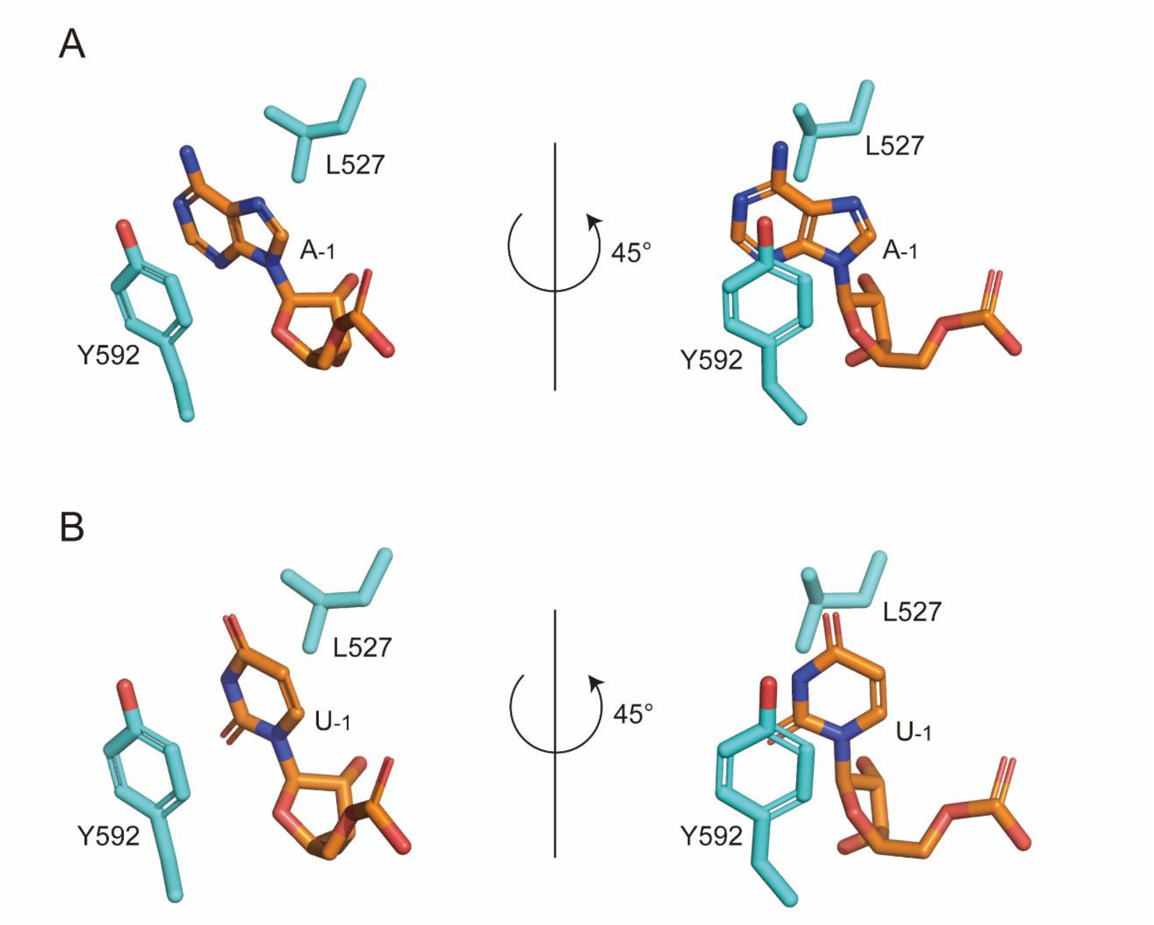

Figure S3. Simple replacement of A_{-1} (A) base with that of uridine (B) in our complex structure show that no hydrophobic stacking interactions are formed between protein and RNA at the -1 nucleotide binding site.

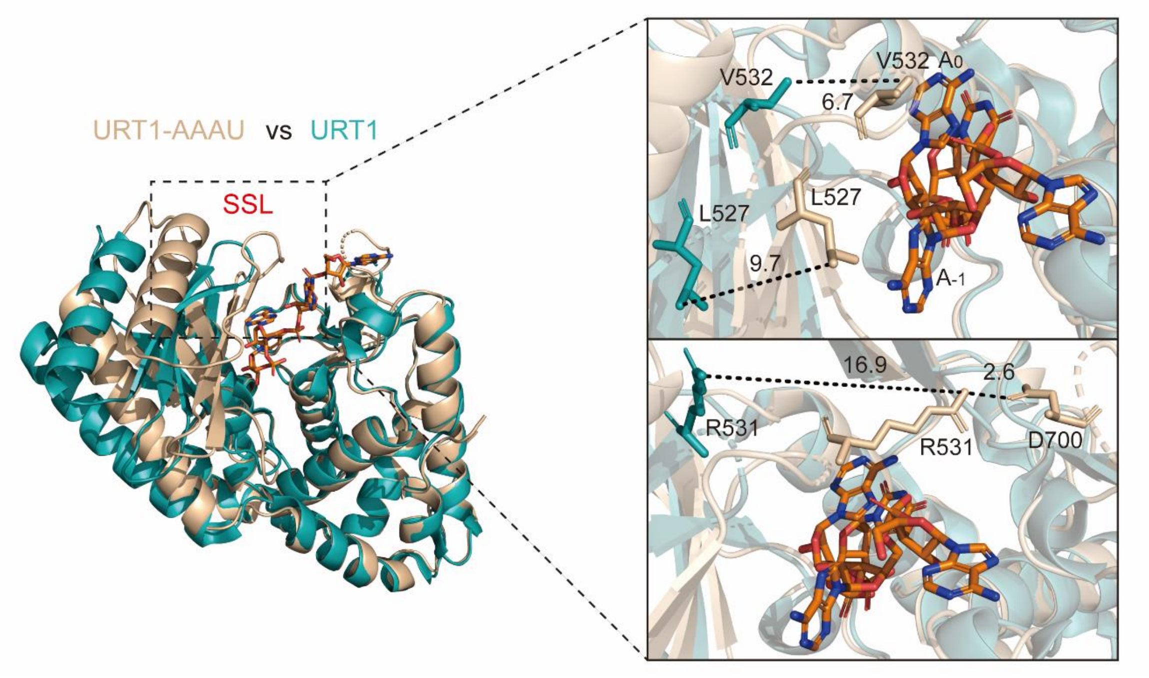

Figure S4. The structural superimposition of apo-form (teal) and RNA-bound (wheat) URT1. (*Inset*) Close-up view of the conserved SSL loop of catalytic domain which undergoes a local structural rearrangement.

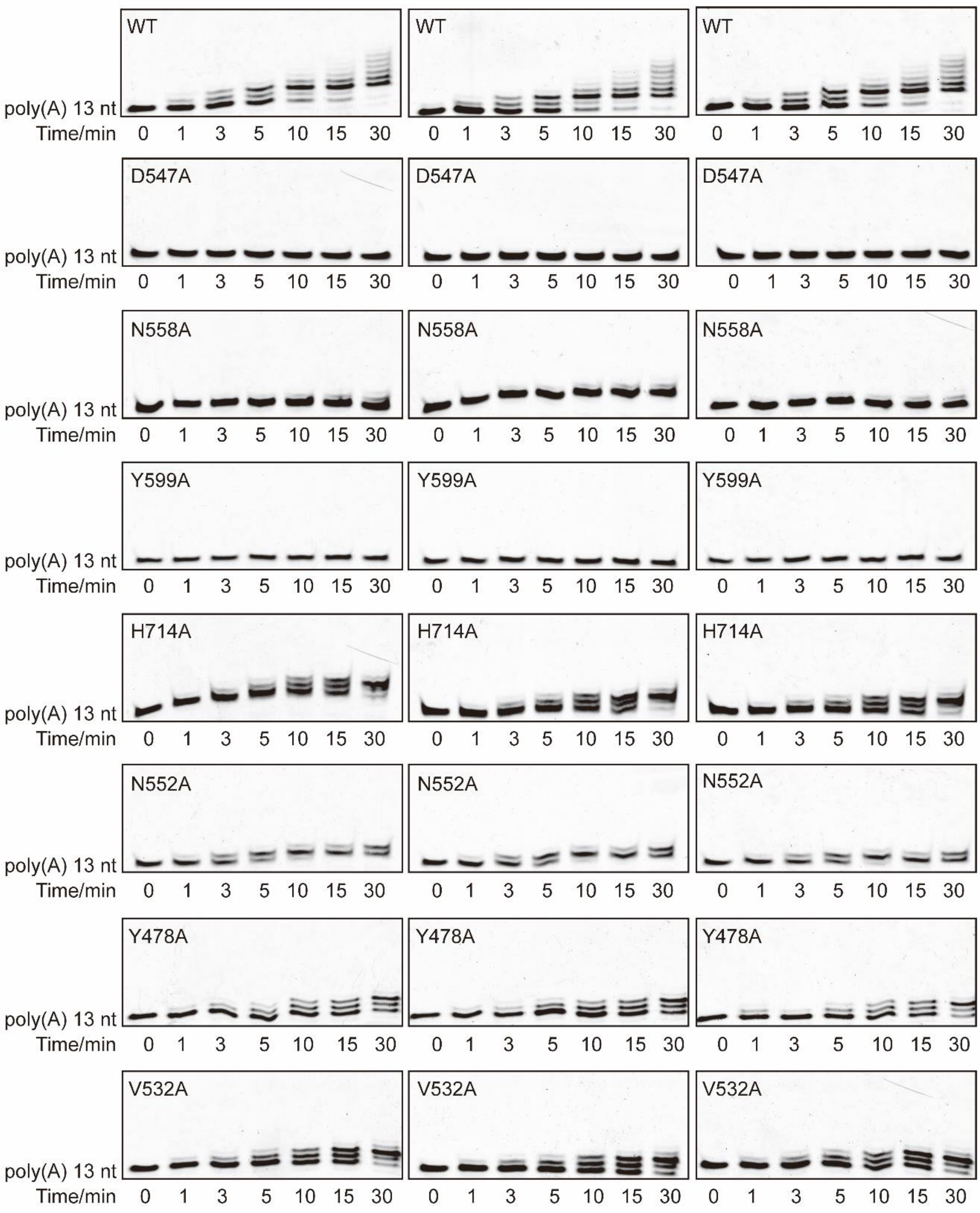

WT
WT
WT
poly(A) 13 nt
Time/min 0 1 3 5 10 15 30
D547A
D547A
D547A
poly(A) 13 nt
Time/min 0 1 3 5 10 15 30
N558A
N558A
N558A
poly(A) 13 nt
Time/min 0 1 3 5 10 15 30
Y599A
Y599A
Y599A
poly(A) 13 nt
Time/min 0 1 3 5 10 15 30
H714A
H714A
H714A
poly(A) 13 nt
Time/min 0 1 3 5 10 15 30
N552A
N552A
N552A
poly(A) 13 nt
Time/min 0 1 3 5 10 15 30
Y478A
Y478A
Y478A
poly(A) 13 nt
Time/min 0 1 3 5 10 15 30
V532A
V532A
V532A
poly(A) 13 nt
Time/min 0 1 3 5 10 15 30

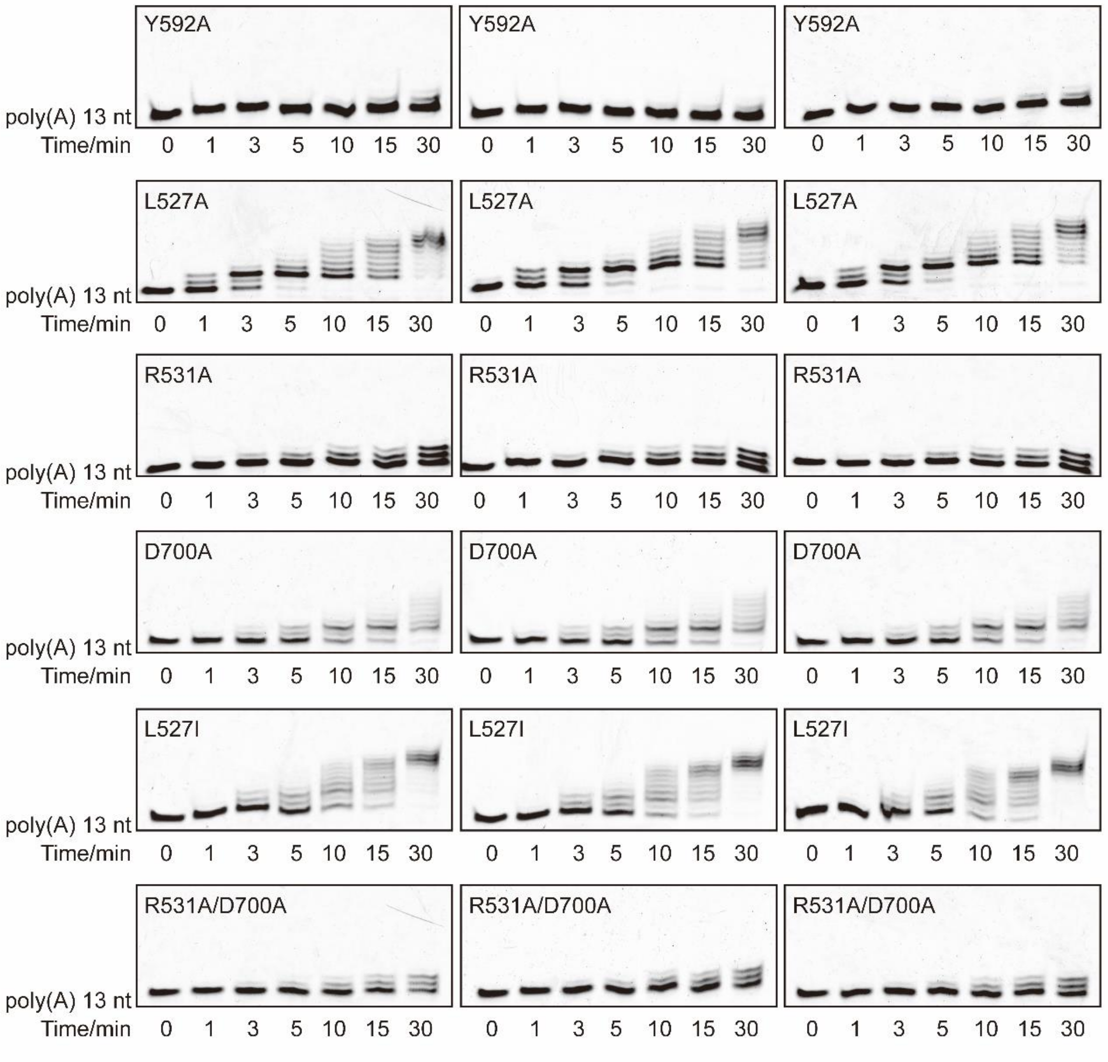

Figure S5. In vitro nucleotide transferase assay of poly(A) by wild-type URT1 and different mutants. Each assay was repeated for three times.

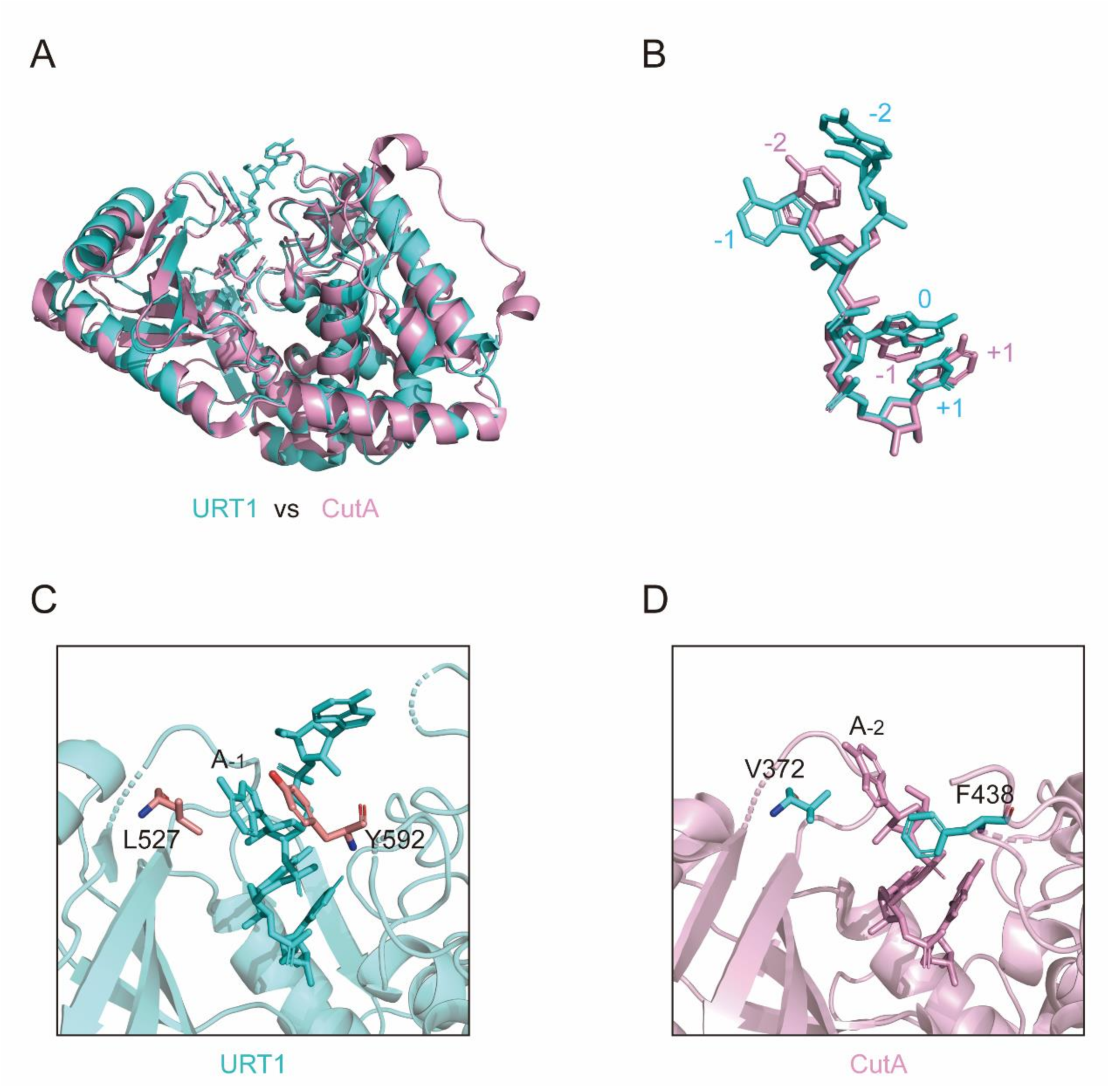

Figure S6. (A) The structural superimposition of URT1-AAAU (cyan) and CutA-AAA (PDB ID 6YWO, pink). (B) The structural superimposition of 5'-AAAU-3' (cyan) and 5'-AAA-3' (pink). Nucleotides are shown in sticks. (C) Detailed diagram of the interaction of nucleotide at the -1 position with surrounding residues in the URT1-AAAU. (D) Detailed diagram of the interaction of nucleotides at the same position with surrounding residues in the CutA-AAA.

Supplementary Methods and Materials

Conventional molecular dynamics (cMD) simulations

cMD simulations were carried out for apo-form URT1, apo-form URT1(R531A), URT1-AAAU complex and URT1(R531A)-AAAU complex by the Amber20 package [1]. Each system was built in the tleap module [2] using the ff14SB force field [3] for protein and bsc0χOL3 force field for RNA [4, 5]. The structures were immersed into a truncated octahedral box that extended 12 Å away from the solute border, using the TIP3P water model [6] and periodic boundary conditions. The ions were added in the box to reach a salt concentration of 300 mM NaCl. Therefore, the total number of atoms was 46,468, 46,683, 44,036 and 44,167, respectively, in the aforementioned systems. The waters and ions were initially minimized for 2000 steps using the steepest descent method for the first 1000 steps and then the conjugate gradient algorithm for the last 1000 steps, with the position of protein and ligands fixed (force constant was 500 kcal mol^{-1} $Å^{-2}$). In the second energy minimization stage, the restraints on the protein and ligands were removed. This stage was conducted for 2500 steps, using the steepest descent method in the first 1000 steps and then the conjugate gradient algorithm for the last 1500 steps. After that, a heat-up MD was run at a constant volume. The system was heated from 0 to 300 K for 100 ps with a weak restraint of 10 kcal mol^{-1} $Å^{-2}$ on the solute. Then, free MD simulations were carried out under the isothermal-isobaric (NPT) condition. Temperature was regulated using the Langevin dynamics [7, 8] with a collision frequency of 1.0 ps^{-1}. Pressure was controlled with isotropic position scaling at 1 bar with a relaxation time of 2.0 ps. All of the bonds involving hydrogen atoms were constrained using the SHAKE algorithm [9]. A 2 fs integration step was used. The long-range electrostatic interactions were calculated using PME method [10] with a 10 Å cutoff for the range-limited non-bonded interactions. Three independent 100-ns cMD simulations were performed for each system.

Accelerated molecular dynamics (aMD) simulations

aMD simulations enhance conformational sampling of a biomolecule by adding a boost

potential $\Delta V_{(r)}$ to the original potential $V_{(r)}$ when the latter is below a threshold energy E [11].

$$\begin{cases} V^*_{(r)} = V_{(r)} & V_{(r)} \gg E \\ V^*_{(r)} = V_{(r)} + \Delta V_{(r)} & V_{(r)} < E \end{cases} \quad (1)$$

In the simplest form, the boost potential is given by

$$\Delta V_{(r)} = \frac{(E-(V_{(r)}))^2}{\alpha + E - V_{(r)}} \quad (2)$$

which can flat the energy potential surface and induce the conformational transition between the low-energy states when the acceleration factor α decreases.

Boosting potentials are often applied to both the total potential and the dihedral energy terms. Here, we used 100 ns cMD trajectories to estimate the aMD input parameters. For apo-form URT1 with 331 residues, the average total potential energy is -156,150 kcal mol^{-1} and the average dihedral energy is 4341 kcal mol^{-1}. The following parameters were set based on the above information:

E(tot) = -156150 kcal mol^{-1} + (0.16 kcal mol^{-1} $atom^{-1}$ × 46468 atoms) ≈ -148716 kcal mol^{-1}

α(tot) = (0.2 kcal mol^{-1} $atom^{-1}$ × 46468 atoms) ≈ 9294 kcal mol^{-1}

E(dih) = 4341 kcal mol^{-1} + (3.5 kcal mol^{-1} $residue^{-1}$ × 331 residues) ≈ 5500 kcal mol^{-1}

α(dih) = 0.2 × (3.5 kcal mol^{-1} $residues^{-1}$ × 331 residues) ≈ 232 kcal mol^{-1}

For the URT1-AAAU with 337 residues, the average total potential energy is -105,482 kcal mol^{-1} and the average dihedral energy was 4441 kcal mol^{-1}. The aMD parameters were set as follow.

E(tot) = -105482 kcal mol^{-1} + (0.16 kcal mol^{-1} $atom^{-1}$ × 44036 atoms) ≈ -98437 kcal mol^{-1}

α(tot) = (0.2 kcal mol^{-1} atom-1 × 44036 atoms) ≈ 8807 kcal mol^{-1}

E(dih) = 4441 kcal mol^{-1} + (3.5 kcal mol^{-1} $residue^{-1}$ × 337 residues) ≈ 5620 kcal mol^{-1}

α(dih) = 0.2 × (3.5 kcal mol^{-1} $residues^{-1}$ × 337 residues) ≈ 236 kcal mol^{-1}

For apo-form URT1(R531A), the average total potential energy is -151,213 kcal mol^{-1} and the average dihedral energy was 4338 kcal mol^{-1}. The aMD parameters were set as

follow.

E(tot) = -151213 kcal mol^{-1} + (0.16 kcal mol^{-1} $atom^{-1}$ × 46683 atoms) ≈ -143744 kcal mol^{-1}

α(tot) = (0.2 kcal mol^{-1} atom-1 × 46683 atoms) ≈ 9337 kcal mol^{-1}

E(dih) = 4338 kcal mol^{-1} + (3.5 kcal mol^{-1} $residue^{-1}$ × 331 residues) ≈ 5497 kcal mol^{-1}

α(dih) = 0.2 × (3.5 kcal mol^{-1} $residues^{-1}$ × 331 residues) ≈ 232 kcal mol^{-1}

For URT1(R531A)-AAAU complex, the average total potential energy is -143,076 kcal mol^{-1} and the average dihedral energy was 4443 kcal mol^{-1}. The aMD parameters were set as follow.

E(tot) = -143076 kcal mol^{-1} + (0.16 kcal mol^{-1} $atom^{-1}$ × 44167 atoms) ≈ -136010 kcal mol^{-1}

α(tot) = (0.2 kcal mol^{-1} atom-1 × 44167 atoms) ≈ 8833 kcal mol^{-1}

E(dih) = 4443 kcal mol^{-1} + (3.5 kcal mol^{-1} $residue^{-1}$ × 337 residues) ≈ 5623 kcal mol^{-1}

α(dih) = 0.2 × (3.5 kcal mol^{-1} $residues^{-1}$ × 337 residues) ≈ 236 kcal mol^{-1}

All the other parameters were the same as those in the cMD simulations. The aMD simulations were performed starting from the final structure of the heat-up procedure, that is to say, the initial conformations of aMD and cMD are the same. To obtain statistically more meaningful results, three independent 2-μs aMD simulation were run for each system.

References

1. Pearlman, D.A., et al., *Amber, a package of computer-programs for applying molecular mechanics, normal-mode analysis, molecular-dynamics and free-energy calculations to simulate the structural and energetic properties of molecules.* Computer Physics Communications, 1995. **91**: p. 1-41.
2. Case, D.A., et al., *The Amber biomolecular simulation programs.* J Comput Chem, 2005. **26**(16): p. 1668-88.
3. Tian, C., et al., *ff19SB: Amino-Acid-Specific Protein Backbone Parameters Trained against Quantum Mechanics Energy Surfaces in Solution.* J Chem Theory Comput, 2020. **16**(1): p. 528-552.
4. Cornell, W.D., et al., *A Second Generation Force Field for the Simulation of*

Proteins, Nucleic Acids, and Organic Molecules. J. Am. Chem. Soc., 1995. **117**: p. 5179-5197.

5. Zgarbova, M., et al., *Refinement of the Cornell et al. Nucleic Acids Force Field Based on Reference Quantum Chemical Calculations of Glycosidic Torsion Profiles.* J Chem Theory Comput, 2011. **7**(9): p. 2886-2902.
6. Mark, P. and L. Nilsson, *Structure and Dynamics of the TIP3P, SPC, and SPC/E Water Models at 298 K.* J. Phys. Chem. A., 2001. **105**: p. 9954-9960.
7. Feller, S.E., et al., *Constant pressure molecular dynamics simulation: The Langevin piston method.* The Journal of Chemical Physics, 1995. **103**(11): p. 4613-4621.
8. Pastor, R.W., B.R. Brooks, and A. Szabo, *An analysis of the accuracy of Langevin and molecular dynamics algorithms.* Molecular Physics, 2006. **65**(6): p. 1409-1419.
9. Forester, T.R. and W. Smith, *SHAKE, Rattle, and Roll: Efficient Constraint Algorithms for Linked Rigid Bodies (vol 19, pg 102, 1998).* J. Comput. Chem., 2000. **21**: p. 157-157.
10. Darden, T., D. York, and L. Pedersen, *Particle mesh Ewald: AnN ·log(N) method for Ewald sums in large systems.* The Journal of Chemical Physics, 1993. **98**(12): p. 10089-10092.
11. Hamelberg, D., J. Mongan, and J.A. McCammon, *Accelerated molecular dynamics: a promising and efficient simulation method for biomolecules.* J Chem Phys, 2004. **120**(24): p. 11919-29.
